# Case of Absence of the Uterus and Consequent Amenorrhœa, with Maltreatment of the Patient

**Published:** 1873-07

**Authors:** Alfred T. Livingston


BUFFALO

### Medical and Surgical Journal.

VOL XII. JULY, 1873. No. 12

### Original Communications.

:o:

ART. I.—*A Case of Absence of the Uterus and consequent Amenorrhœa, with Maltreatment of the patient.* By ALFRED T. LIVINGSTON, M. D.

For permission to publish the history of the following case, I am indebted to Prof. JAMES P. WHITE, under whose charge the case came, and through whose kindness I had repeated opportunities for its observation.

The case possesses sufficient interest as an anomaly to warrant its publication, and taken in connection with its practical relation to the diagnosis and treatment of Amenorrhœa, it is doubly interesting and important.

Mrs. B. is now thirty years of age and has been married six years.

While contemplating marriage, a few months previous to that event, *having never menstruated,* she consulted a doctor in reference to this fact.

This person, Dr. V. S. examined her *simply per vaginam,* and informed her that she had occlusion of that organ. He proceeded, at once to operate for the relief of her trouble, by making an incision, through what he supposed no doubt, to be the occluding membrane—an imperforate hymen.

He assured her that she possessed the organs of her sex in completeness; and the hemorrhage, that occurred after the incision, was stated to be the retained catameniæ.

On the assurance of the doctor, that she was a perfect woman, she married, six months after the operation.

During the six months intervening between the operation and her marriage, the patient observed that in micturition, all the urine did not come from the normal outlet of the urethra, but that a portion came down the vagina.

The urine did not flow from this new channel except when she urinated voluntarily, so that this urethro vaginal fistula, for such it must have been, did not give her especial trouble or discomfort.

Soon after marriage, however, she began to have non-retention of urine. She was then obliged to wear constantly a sponge, napkin, or other appliance for the purpose of catching the dribbling urine, While about upon her feet the urine flowed away as fast as secreted, but when lying down it was retained for a time only to gush forth, *en masse,* on the instant of rising.

Now, this change in symptoms, following marriage, can only be accounted for by supposing that the introduction of the male organ, either extended the fistulous opening, which the doctor had made through the urethro-vaginal wall, so that it involved the vesico-vaginal wall, thus becoming a urethro-vesico-vaginal fistula; or, more gradually, dilated both the fistula and the portion of urethra between the fistula and base of the bladder; and so largely and thoroughly dilated them, that, with the more or less frequent sexual intercourse ever since, they have not been allowed to contract. It is difficult to say precisely, which of these two things occurred; and it is of no consequence except, that if the latter supposition be correct, the patient will more probably, in fact, almost certainly regain complete control over the sphincter vesicæ,—in other words —perfect retention of urine, when time and cessation from the act of coition will have allowed the upper portion of the urethra to contract.

The complete incontinence of urine, following marriage, continued, with all its distressing symptoms and accompaniments, until a recent date.

Desirous, as she well might be, of relief, Mrs. B. about a year ago, consulted another physician, Dr. P., of Michigan.

Upon examination he informed her, that, in consequence of its continued idleness, the urine having, for so long a time passed through an abnormal channel, *the urethra had closed;* its walls, being in contact, had grown together.

Now, besides the great improbability of such an occurrence, there is very good evidence that it did not take place, as we shall presently see.

But Doctor P. thought it best to open (?) the natural passage, before attempting to close the false one; and this he did, it appears, by dividing with an incision the portion of the urethro-vaginal wall that yet remained, viz.: from the orifice of the urethra back to the fistulous opening.

Greatly dissatisfied with *this one,* Mrs. B. did not allow the Dr. to make his proposed *second* operation; but selected Dr. WHITE as the one to whom she would look for relief from the condition that rendered her life so miserable.

She accordingly came to Buffalo in October, 1872, and was then operated on by Dr. White, assisted by Drs. Rochester, Potter, and myself.

The patient was a fair and delicate person of slight build and medium stature, possessing the mammæ well developed, and the external genitalia perfect. The vagina, however, was a mere *cul de sac,* little more than an inch in length. Proper examination discovered, also, the presence of the ovaries and the *entire absence* of the *uterus.*

The urethra and vagina, as far as to the summit of the latter, had been converted into a single tube, by the incisions through the intervening wall, made as above described.

This urethro-vagina was connected with the interior of the bladder by a large but short canal, which was either the neck of the bladder, or the vesical end of the urethra, greatly dilated as before suggested.

A deep groove, smooth and lined with mucus membrane, occupying the anterior wall of the common canal just mentioned, was the remains of the urethra and the smoothness and gloss of its surface, indicated clearly enough, that the urethra had never been *closed,* as Dr. P. asserted.

The operation by Dr. WHITE was made, by carefully paring the edges of this divided urethro-vaginal wall and bringing the freshly cut surfaces together, by means of seven or eight interrupted silver sutures.

A portion of the surfaces failed to unite on account of pressure, made by the catheter used in the after-treatment, against the lips of the wound. It was made necessary by this, to repeat the operation, in part, which was done some three weeks subsequently.

The most important lesson we learn from the history of this unfortunate person, is the care that should be exercised in determining the *cause* of Amenorrhœa before resorting to treatment. *Occlusion* of the vagina should be distinguished from *absence* of the vagina, or the uterus, or the ovaries.

Had Dr. V. S., (it is due to the profession to say, that he is a Quack,) properly examined his patient, it would have been an easy matter to explain the absence of menstruation and it would likewise have saved her many years of no little suffering, which his treatment (!)induced; not to speak of the embarrassing and unhappy position, in which his untrue decision has placed both the patient and her husband.

The position of the uterus being between the bladder, in front, and the rectum, behind, its presence or absence is readily determined.

If a sound or catheter be introduced into the bladder, it will be distinctly felt by the finger passed up the rectum,—*provided* the uterus be absent; and will not be so felt if present. Again, if the finger in the rectum plainly detects the other hand placed upon the hypogastrium, the absence of the uterus may be inferred; and, if to these tests be added that of examination per vaginam, with indications in each instance of the absence of the uterus, no doubt need be entertained of the certainty of that condition. The presence or otherwise of the ovaries may be concluded upon, by seeking for them with the finger of one hand well up in the vagina or, better, in the rectum, and the other hand placed over the right and left iliac regions respectively. In the patient we are considering, the absence of the uterus and the presence of the ovaries were determined upon only, after the examinations I have indicated.

The existence of ovaries of normal size, together with the perfect development of the mammæ and external genitalia, referred to above, would lead us to suspect the occurrence of a menstrual molimen, and this we find to obtain. At regular intervals of about a month, a sufficient number of the symptoms of menstruation occur to indicate their character and cause, although no sanguinolent discharge takes place, for the obvious reason that there is no secreting surface; the menstrual hemorrhage, as is well known, taking place from the lining membrane of the body and fundus of the uterus.

Under these circumstances the existence of a vicarious hemorrhage would suggest itself as quite possible, but this has not been observed.

There exists, moreover, in this patient the venereal appetite and coition is attended with pleasure, although every introduction of the penis has been directly into the bladder.

This contact of a foreign body with the mucous membrane of the bladder very naturally induced cystitis, which became chronic. The purulent discharge that occurred was called Leucorrhœa and various astringent injections were used but without giving relief.

The bladder, owing to the perfectly free exit of the urine from its cavity, has become very firmly contracted and will but gradually return to its normal dilatability.

The patient can now retain her urine quite well during two or three hours; and the prospect is good for her final perfect recovery.

